# Supplementary material for: Single-Cell Analysis of Host Responses in Bovine Milk Somatic Cells (bMSCs) Following HPAIV Bovine H5N1 Influenza Exposure
Source: Viruses. 2025 Jun 3;17(6):811. doi: 10.3390/v17060811 (PMC12197475; doi:10.3390/v17060811)
Supplement: Supplementary file 1 [file viruses-17-00811-s001.zip › Supplementary Figure.pdf]

Supplementary Figure

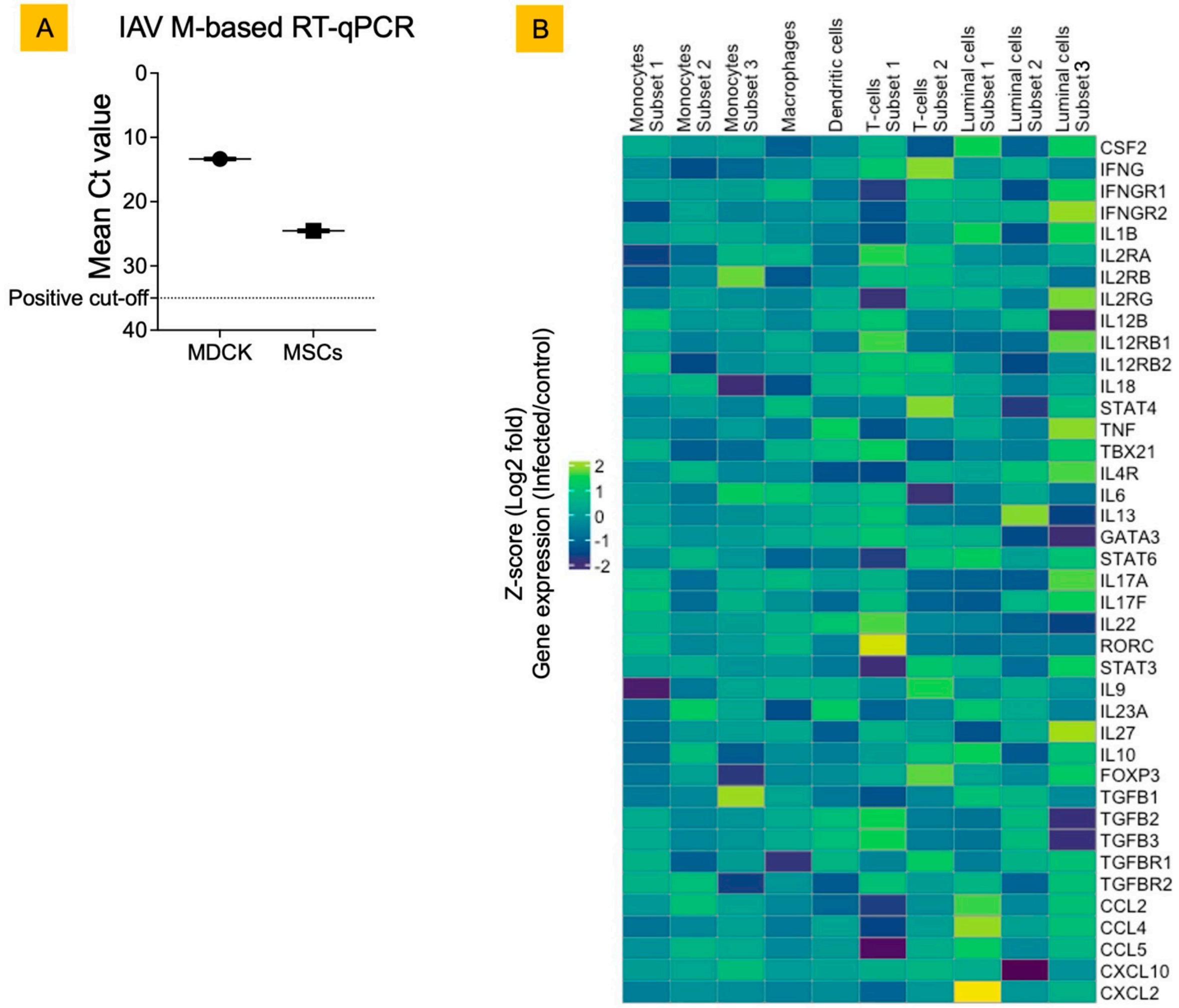

Supplementary figure S1: Immune response and viral detection in bovine MSCs infected with bovine-H5N1. (A) The dot plot shows the results of IAV M-gene-based RT-qPCR in MDCK cells (positive control) and bMSCs infected with bovine-H5N1. The x-axis represents the mean Ct values, and y-axis represents the cell type. (**B)** A heatmap of gene expression of various immune-related genes as z-scored log2 fold-change after bovine-H5N1 infection in different cell clusters.
